# Inoculation of Medicines

**Published:** 1848-05

**Authors:** 


*Inoculation of Medicines.*—Dr. Lefargue proposes for the introduction of drugs into the system, a method whicd had not hitherto been thought of, viz., inoculation.

1. *Narcotics.*—If a lancet, loaded at its point with a salt of morphia reduced to a paste by the addition of some water, be introduced horizontally under the epidermis, a slight swelling and trifling congestion immediately occur, accompanied by a sense of heat and some pruritus. The morphia is evidently absorbed in a very short space of time, headache, yawning; and dryness of the mouth making their appearance. This method applies particularly to neuralgia of the face and scalp, and causes neither pain nor scars. Inoculation gives instant relief from toothache (inoculation of the gum,) from the pains which follow herpes zoster, &c.

2. *Solaneæ.*—The extracts of belladonna and of hyoscyamus are too weak to be of much avail when introduced by inoculation with the lancet; but if numerous punctures be made upon any part of the integument, and a thick solution of these extracts is applied, the absorption is active and efficient.

3. *Strychnia.*—When this substance is introduced into the system by inoculation, its effects are very prompt, it relieves partial paralysis, and has been successful in rebellious chorea when inoculated along the spine.

4. *Veratrine* calms rapidly facial neuralgia and obstinate headache.

5. *Croton oil and tartar emetic* have also been employed in the same manner for the purpose of producing revulsion.—*Ibid.*

